# Neighbourhood deprivation and very preterm birth in an English and French cohort

**DOI:** 10.1186/1471-2393-13-97

**Published:** 2013-04-25

**Authors:** Mercedes Bonet, Lucy K Smith, Hugo Pilkington, Elizabeth S Draper, Jennifer Zeitlin

**Affiliations:** 1 INSERM, UMR S953, Epidemiological Research Unit on Perinatal Health and Women’s and Childrens’ Health, Paris, France; 2 UPMC, Univ Paris 06, Paris, France; 3 Department of Health Sciences, University of Leicester, Leicester, LE1 6TP, UK; 4 Department of Geography, University Paris 8 Vincennes-Saint Denis, Saint Denis, France

**Keywords:** Very preterm infants, Social inequalities, Europe, Census data

## Abstract

**Background:**

Social factors affect the risk of very preterm birth and may affect subsequent outcomes in those born preterm. We assessed the influence of neighbourhood socio-economic characteristics on the risk and outcomes of singleton very preterm birth (<32 weeks of gestation) in two European regions with different health systems.

**Methods:**

Live births (n=1118) from a population-based cohort of very preterm infants in 2003 in Trent (UK) and Ile-de-France (France) regions were geocoded to their neighbourhood census tracts. Odds ratios for very preterm singleton birth by neighbourhood characteristics (unemployment rate, proportion manual workers, proportion with high school education only, non home ownership) were computed using infants enumerated in the census as a control population. The impact of neighbourhood variables was further assessed by pregnancy and delivery characteristics and short term infant outcomes.

**Results:**

Risk of very preterm singleton birth was higher in more deprived neighbourhoods in both regions (OR between 2.5 and 1.5 in the most versus least deprived quartiles). No consistent associations were found between neighbourhood deprivation and maternal characteristics or health outcomes for very preterm births, although infants in more deprived neighbourhoods were less likely to be breastfed at discharge.

**Conclusions:**

Neighbourhood deprivation had a strong consistent impact on the risk of singleton very preterm birth in two European regions, but did not appear to be associated with maternal characteristics or infant outcomes. Differences in breastfeeding at discharge suggest that socio-economic factors may affect long term outcomes.

## Background

Preterm birth is related to individual social factors, including maternal education, occupation and ethnicity [1,2], as well as neighbourhood social characteristics [1,3]. The influence of these factors seems to be greater for very preterm births (<32 weeks) than for moderately preterm births (32-36 weeks) [4-7]. Rates of preterm birth have increased steadily in recent decades in Europe [6-10] and social disparities have persisted over time [6,7,9].

Mechanisms underlying the association between the risk of very preterm birth and social factors are not well understood [11]. Attempts to identify distinct social risk factors for subtypes of preterm births, including those associated with spontaneous very preterm labour [12], specific maternal complications of pregnancy [13-15] or multiple pregnancies [16,17], have yielded contradictory findings. Preterm birth has been also associated with a greater burden of neonatal care [18], but little research has been carried out on whether social factors are related to very preterm infant outcomes before discharge from the neonatal unit [19].

Wide variations in very preterm births rates exist in Europe (i.e. from 0.9% to 1.4% of total live births in France and England) [20]. Research suggests that the relationship between socio-economic deprivation and high risk births differ slightly across developed countries. For instance, the increased risk of very preterm birth in more deprived compared to less deprived neighbourhoods varied between 1.6 [7] and 1.9 [9] in the UK, and a two-fold risk for low birth-weight births(<2500 grams) in the US [21]. These two countries are known to have high rates of very preterm births when compared to other developed countries [20]. Cross-national comparisons make it possible to investigate whether the impact of social factors is more acute in contexts where preterm birth rates are higher. More generally, these comparisons enable exploration of variations in the social gradient in risk of preterm birth that may depend on policy or population factors [6,22].

Cross-national comparisons of the impact of social factors on the risks and outcomes of very preterm birth have been limited because of the difficulties of defining comparable populations as well as the lack of socioeconomic information in studies on these infants. International variations in procedures for recording births and deaths at early gestational ages [23] as well as the assessment of gestational age make it difficult to compare populations of very preterm births unless a common inclusion protocol is used. Second, most data on very preterm births come from cohort studies [24-26] or hospital registers [27] and these do not usually include a comparison group of term births which is needed to assess the risk of preterm birth associated with social factors. Finally these studies are often based on data abstracted from medical records which contain limited information on individual social characteristics. While area-based measures offer a solution in this situation, common indicators are required; area-based measures may not be comparable because of the use of different indicators or scores of social disadvantage.

We investigated the influence of neighbourhood socio-economic status on the risk of very preterm singleton birth in two French and English regions with different very preterm rates and health systems using data from the population-based MOSAIC study collected with a common protocol and standardized inclusion and validation criteria. A second aim was to study whether the clinical characteristics of pregnancy, very preterm delivery, in-hospital mortality and short term infant health outcomes varied by neighbourhood of residence.

## Methods

### Very preterm infants

Data were available for very preterm infants born between 22+0 to 31+6 weeks from the former Trent health region (UK) and Ile-de-France (France), two regions participating in the population-based MOSAIC study [28]. The overall very preterm live birth rate in the cohort was significantly higher in Trent (13.0/1000 live births) than in Ile-de-France (10.2/1000 live births) [28]. Ethics approval was sought for the collection of these data as required in each of the regions. Local research ethics committee approval was obtained for each of the centres in the Trent region. Authorisation for the constitution of the MOSAIC database in conformity with data confidentiality laws in France was provided by the CNIL (Commission Nationale Informatique et Libertés) on March 2003 (no. 03-1052).

Data were collected on all very preterm births from 01/01/2003 to 31/12/2003 in Trent and from 01/02/2003 to 31/08/2003 in Ile-de-France. Information on maternal and infant’s characteristics were abstracted from medical records using a common protocol. Infants were followed until discharge from hospital (to home or long-term care) or death. Inclusions in the study were cross-checked with birth registers in each maternity unit. After excluding fetal deaths before onset of labour and infants born or resident outside the study regions, 667 infants in Trent (534 singletons) and 809 in Ile-de-France (584 singletons) were available for analysis.

Information on characteristics of the very preterm delivery included gestational age, small for gestational age, multiplicity (singleton or twin), indicated delivery (caesarean section before labour) and pregnancy complications: hypertension (pregnancy-induced hypertension, pre-eclampsia, eclampsia, and HELLP syndrome); antepartum haemorrhage without hypertension; preterm premature rupture of membranes (PPROM) without hypertension or haemorrhage; and preterm labour without hypertension, haemorrhage or PPROM. Infant outcome measures included in-hospital mortality, mechanical ventilation (>1 day), long hospital stay (longer hospital stay than 75^th^ percentile for gestational age), bronchopulmonary dysplasia and neurological morbidity (intraventricular haemorrhage grade III and IV or cystic periventricular leukomalacia). We also included breast milk feeding at discharge. Definitions have been published previously [28,29].

No data were routinely available on maternal socio-economic factors from medical records and so information on the location of mother’s place of residence was collected. Subsequent analysis on the effects of social factors on very preterm birth and health outcomes were carried out in Ile-de-France and the Trent region, as both countries could provide census data on small geographic areas using similar data collection methodology. These data were thus used to characterize neighbourhoods of residence for very preterm infants. Very preterm infants were geocoded to the smallest geographic units for which census data were available based on their mothers’ place of residence: super output areas in Trent (approximately 1500 inhabitants and a median surface of 72.4 Km^2^) and the IRIS-2000 in Ile-de-France (between 1800 to 5000 inhabitants and a median surface of 70.0 Km^2^).

### Census population

Census data were used to constitute a comparison group of all infants living in each region, because data on all births were not available by census areas for all the Ile-de-France districts.

We obtained census information on the number of individuals aged “0” in the census closest to the date of our cohort in order to estimate the population of recent births by neighbourhood of residence in each region. Individuals aged “0” in the census correspond to babies born between the first of the year and the final day of the census (from 01/01/2001 to 04/29/2001 (N=18,477) in Trent and from 01/01/1999 to 03/08/1999 (N=21,405) in Ile-de-France).

Sensitivity analyses were carried out to test that the census accurately reflected the birth cohort’s place of residence, using birth registries for the whole 2003 year in Trent and from one of the districts in 1999 in Ile-de-France.

### Study sample

Analyses were restricted to singleton very preterm births as we could not differentiate births by multiplicity in the denominator from the census data. Rates of very preterm singleton births were similar in the two regions (80.9% in Trent and 80.2% in Ile-de-France). Multiples are only 3% of all births and tend to have different characteristics from total births, including older more affluent mothers. Furthermore, the sample was small (133 twins in the Trent region and 225 twins in Ile-de-France) and this sample was inadequate for twin specific analysis which are necessary as maternal complications and outcomes differ for twins.

### Neighbourhood socio-economic characteristics

We studied four neighbourhood socio-economic characteristics (Table 1) (unemployment rate, proportion of manual workers, proportion with a high school education only, and proportion of non home owners) which were selected based on the literature and the comparability of census definitions. Neighbourhoods were grouped into quartiles by each socio-economic characteristic based on the place of residence of infants enumerated in the census within each region (1=least deprived to 4=most deprived). Very preterm infants were then assigned to the appropriate quartile for each indicator according to their mothers’ place of residence. We studied each indicator separately because no validated common score exists for these regions.

**Table 1:** Census area socio-economic measures

**Neighbourhood characteristics**	**England**		**France**	
	**Definition (ONS)**	**Median**	**Definition (INSEE)**	**Median**
		**(IQR)**		**(IQR)**
Unemployment rate	Unemployed: A person (16-74) is defined as unemployed if he or she is not in employment, is available to start work in the next 2 weeks and has either looked for work in the last 4 weeks or is waiting to start a new job. This is consistent with the International Labour Office (ILO) standard classification	3.2 (2.1-5.1)	Ratio between the number of unemployed (those who declared themselves "unemployed", or not registered at the ANPE (unemployment office), unless they have explicitly said they are not looking for work) and the working population (15 and over)	10.9 (8.3-15.7)
Proportion of manual workers	NS-SEC: employees in semi-routine occupations (sales, service, technical, operative, agricultural, clerical and child-care) and routine occupations (sales, service, production, technical, operative, agricultural).	26.4 (19.0-32.2)	Employees in skilled occupations (industrial, craft workers, drivers, handling, warehousing and transportation) or unskilled occupations (unskilled industrial, unskilled craft workers) and agricultural occupations	10.4 (5.75-16.0)
Proportion of residents with less than high school	People aged 16 to 74 with no academic qualifications, or level 1 or level 2 highest qualification.Level 1: one or more O levels/ Certificate of Secondary Education (CSE)/General Certificate of Secondary Education (GCSE) (any grade); National Vocational Qualification (NVQ) level 1; Foundation General National Vocational Qualification (GNVQ)	72.4 (63.6-79.0)	People aged 15 or more with no academic qualifications, or with ‘Certificat d'Aptitudes Professionelles’ (CAP) or ‘Brevet d'Etudes Professionnelles’ (BEP) highest qualification.	59.7 (44.2-72.3)
	Level 2: five or more CSEs (grade 1); five or more GCSEs (grade A – C); one or more A levels/AS levels; NVQ level 2; Intermediate GNVQ			
Proportion of non home owners	Home owner includes accommodation that is either owned outright, owned with a mortgage or loan, or shared ownership (paying part rent and part mortgage).	27.4 (13.9-47.2)	Owner or co-owner households include different forms of home ownership (owned outright, accommodation owned with a mortgage or loan)	63.0 (45.6-76.0)

Median and IQR (interquartile range) in the population of infants 0 years enumerated in the census; INSEE: National Institute for Statistics and Economic Studies; ONS: Office for National Statistics; NS-SEC: The National Statistics Socio-economic Classification.

### Statistical analysis

We first compared singleton very preterm infants with census infants by neighbourhood characteristics quartiles within each region. Odds ratios (OR) with 95% confidence intervals were calculated for the risk of very preterm birth; we tested for trends across deprivation quartiles and for interactions between neighbourhood characteristics and region. While we could not exclude multiple births from the census data, these constitute a small fraction of all births and the census population provides a good approximation of the characteristics of the singleton population.

Then pregnancy and delivery characteristics and infant outcomes for very preterm singleton births were compared across the four socio-economic neighbourhood characteristics. Adjusted odds ratios (aOR) were calculated, controlling for maternal age, small for gestational age and sex, using logistic regression models. The small number of infants with brain lesions did not make it possible to calculate aOR for this outcome. We tested whether the association between pregnancy and delivery characteristics and infant outcomes varied by each neighbourhood characteristic between the two regions. Interactions were considered significant if p<0.05. Analyses were undertaken to investigate all four neighbourhood deprivation characteristics. Here associations are shown for unemployment rates alone to simplify the presentation of results.

Data were analysed using STATA 9 software (StataCorp LP, College Station, TX, USA).

## Results

Table 1 shows median and interquartile range values for neighbourhood deprivation characteristics in the census population of babies “0” years of age. Sensitivity analyses comparing the socio-economic distribution of the census population of infants to birth registers yielded similar quartile cut-offs in both regions. Mothers of very preterm singletons were older in the Ile-de-France region than in Trent (Table 2). Gestational age, sex, birthweight and multiplicity were similar across the two regions. The distribution of pregnancy complications and very preterm infant outcomes differed between regions, except for hypertension during pregnancy, caesarean section before labour and in-hospital mortality.

**Table 2:** Characteristics of very preterm singletons in Trent and Ile-de-France

	**Very preterm infants**		
	**Trent**	**Ile-de-France**	**p**
**Characteristics of very preterm population**			
Maternal age [mean (SD)]	27.4 (7.1)	30.3 (6.0)	(<.001)
Gestational age [mean (SD)]	28.3 (2.4)	28.2 (2.5)	(0.8)
Birthweight [mean (SD)]	1162 (407)	1116 (394.5)	(1.0)
Sex [% male]	52.8	52.0	(0.8)
**Characteristics of very preterm pregnancy and delivery**			
Hypertension during pregnancy^a^	20.6	24.9	(0.07)
Antepartum haemorrhage without hypertension	38.8	15.9	(<.001)
Premature rupture of membranes without hypertension or antepartum haemorrhage	13.3	19.7	(0.002)
Preterm labour without hypertension, haemorrhage or PPROM	10.9	14.9	(0.03)
Caesarean section before labour	40.9	44.3	(0.2)
**Very preterm infant outcomes**			
In-hospital mortality^b^	20.0	24.3	(0.06)
Bronchopulmonary dysplasia^c,d^	22.0	15.8	(0.01)
Brain lesions ^c,e^	7.6	11.4	(0.03)
At least one day on ventilator ^c^	60.9	73.0	(<.001)
Long hospital stay^c,f^	44.1	63.3	(<.001)
Breast milk at discharge^c^	35.9	26.9	(0.002)

a) Hypertension during pregnancy: pregnancy-induced hypertension, pre-eclampsia, eclampsia and HELLP syndrome.

b) In-hospital mortality includes death during labour, delivery or hospitalization after birth.

c) Includes only infants discharge alive.

d) Oxygen dependence or ventilation, including nasal continuous positive airway pressure, at 36 weeks of gestational age.

e) Brain lesions includes intraventricular haemorrhage and periventricular leukomalacia.

f) Long hospital stay: longer hospital stay than 75^th ^percentile for gestational age.

In both regions, the percentage of very preterm singletons increased with a higher rate of unemployment, a higher proportion of manual workers and non-home owners or a low educational level when compared to infants in the census (Table 3). More than 30% of the very preterm infants lived in the most deprived quartile compared with around 15% in the least deprived quartile for all four deprivation indicators, with statistically significant odds ratios ranging between 2.5 and 1.5 (test for trend of OR: p<.001) for most deprived compared to least deprived quartiles. There was no evidence of a difference in the magnitude of the effect of deprivation between the two areas (P values for test for interactions ranged between 0.1 and 0.9).

**Table 3:** Distribution and odds ratio of risk of very preterm singleton compared to infants in the census by neighbourhood socioeconomic characteristics

	**Census infants**		**Very preterm singletons**							
**Neighbourhood characteristics quartiles**	**Trent**	**Ile-de-France**	**Trent (n=534)**				**Ile-de-France (n=584)**			
	**N**	**N**	**N**	**% (p)**	**OR**	**95%CI**	**n**	**% (p)**	**OR**	**95%CI**
**Unemployment rate**				(<.001)				(<.001)		
1st (least deprived)	4 670	5 355	84	15.7	1		83	14.2	1	
2nd	4 576	5 346	103	19.3	1.3	(0.9-1.7)	122	20.9	1.5	(1.1-2.0)
3rd	4 625	5 355	140	26.2	1.7	(1.3-2.2)	177	30.3	2.1	(1.6-2.8)
4th (most deprived)	4 606	5 349	207	38.8	2.5	(1.9-3.2)	202	34.6	2.4	(1.9-3.2)
**Proportion of manual workers**				(<.001)				(<.001)		
1st	4 623	5 359	77	14.4	1		80	13.7	1	
2nd	4 620	5 339	136	25.5	1.8	(1.3-2.3)	146	25.0	1.8	(1.4-2.4)
3rd	4 617	5 351	156	29.2	2.0	(1.5-2.7)	182	31.2	2.3	(1.8-3.0)
4th	4 617	5 355	165	30.9	2.2	(1.6-2.8)	176	30.1	2.2	(1.7-2.9)
**Proportion of residents with less than high school**				(<.001)				(<.001)		
1st	4 633	5 352	89	16.7	1		90	15.4	1	
2nd	4 602	5 354	114	21.4	1.3	(1.0-1.7)	137	23.5	1.5	(1.2-2.0)
3rd	4 617	5 349	132	24.7	1.5	(1.1-2.0)	167	28.6	1.9	(1.4-2.4)
4th	4 625	5 350	199	37.3	2.2	(1.7-2.9)	190	32.5	2.1	(1.6-2.7)
**Proportion of non home owners**				(<.001)				(<.001)		
1st	4 626	5 354	95	17.8	1		122	20.9	1	
2nd	4 605	5 351	94	17.6	1.0	(0.8-1.3)	129	22.1	1.1	(0.8-1.3)
3rd	4 629	5 348	150	28.1	1.6	(1.2-2.1)	147	25.2	1.2	(1.2-2.1)
4th	4 617	5 352	195	36.5	2.1	(1.6-2.6)	186	31.9	1.5	(1.6-2.6)

Test for trend of OR: p<.001 for singletons in Trent and Ile-de-France for all neighbourhood characteristics.

Table 4 compares pregnancy and delivery characteristics and infant outcomes for singleton very preterm births by unemployment rate quartiles. Older women tended to live in least deprived neighbourhoods in the Trent region; but no differences were observed in Ile-de-France. There was no evidence of a relationship between neighbourhood unemployment rate and gestational age, small for gestational age, indicated preterm delivery, haemorrhage during pregnancy or preterm labour. Hypertension during pregnancy decreased with neighbourhood deprivation in Trent but increased in Ile-de-France. The percentage of women with preterm premature rupture of membranes tended to be lower in more deprived neighbourhoods in both regions. There was no evidence of variation with neighbourhood deprivation for any of the very preterm outcomes (in-hospital mortality, bronchopulmonary dysplasia, brain lesions, ventilation and length of hospital stay) except for breastfeeding. Results were similar for the three other neighbourhood socio-economic indicators.

**Table 4:** Pregnancy and delivery characteristics and infant outcomes by neighbourhood unemployment rate quartiles (percentages)

	**Trent**							**Ile-de- France**						
		**Unemployment rate quartiles**						**Unemployment rate quartiles**						
	**Cases**	**1**^**st **^**(least)**	**2**^**nd**^	**3**^**rd**^	**4**^**th **^**(most)**	**p**^**a**^	**p**^**b**^	**Cases**	**1**^**st **^**(least)**	**2**^**nd**^	**3**^**rd**^	**4**^**th **^**(most)**	**p**^**a**^	**p**^**b**^
**Characteristics of very preterm pregnancy and delivery**														
Maternal age [mean (SD)], years	533	30.6	28.8	27.4	25.2	(<.001)		584	31.7	30.2	30.4	29.6	(0.07)	
		(5.2)	(6.5)	(9.0)	(5.9)				(5.6)	(5.5)	(6.1)	(6.4)		
Gestational age at delivery						(0.4)							(0.2)	
less ≤27 weeks	175	36.9	28.2	34.3	32.4			205	39.8	23.8	36.2	39.1		
28-29 weeks	139	31.0	29.1	20.7	26.1			160	26.5	32.0	26.6	25.7		
30-31 weeks	220	32.1	42.7	45.0	41.6			219	33.7	44.3	37.3	35.2		
Small for gestational age^c^						(0.3)							(0.6)	
<10 percentile	44	14.1	11.0	5.9	7.3			68	7.7	12.7	16.6	10.8		
10-24^th^	75	10.3	12.0	17.8	16.1			109	20.5	20.3	18.4	21.1		
≥25^th^	387	75.6	77.0	76.3	76.7			367	71.8	67.0	65.0	68.1		
Hypertension during pregnancy^d^	109	31.0	22.3	17.9	16.9	(0.04)	(0.1)	128	19.3	19.7	19.2	26.7	(0.2)	(0.2)
Antepartum haemorrhage without hypertension	218	39.3	42.7	37.1	43.0	(0.7)	(0.7)	95	19.3	16.4	14.7	16.3	(0.8)	(0.8)
Premature rupture of membranes without hypertension or antepartum haemorrhage	66	15.5	11.7	17.9	7.7	(0.03)	(0.02)	118	25.3	16.4	26.0	15.4	(0.03)	(0.03)
Preterm labour without hypertension, haemorrhage or PPROM	56	4.8	8.7	10.0	14.0	(0.1)	(0.2)	95	15.7	16.4	13.0	19.3	(0.4)	(0.5)
Caesarean section before labour	215	40.5	39.8	40.0	40.6	(1.0)	(1.0)	247	39.8	47.5	42.9	39.6	(0.9)	(0.6)
**Very preterm infant outcomes**														
In-hospital mortality^e^	125	19.1	17.5	22.1	20.8	(0.8)	(0.5)	167	33.7	22.0	22.6	26.2	(0.2)	(0.1)
Bronchopulmonary dysplasia^f,g^	86	20.6	23.5	17.4	20.1	(0.8)	(0.5)	67	12.7	16.1	19.3	12.8	(0.5)	(0.5)
Brain lesions^f,h^	26	4.4	9.4	7.3	4.3	(0.4)		19	3.6	2.1	4.4	6.1	(0.8)	
At least one day on ventilator^f^	237	64.0	65.6	63.2	57.7	(0.3)	(0.4)	304	77.9	71.7	75.0	70.4	(0.7)	(0.7)
Long hospital stay^f,i^	108	23.5	32.9	22.0	24.4	(0.5)	(0.4)	108	20.0	25.5	26.3	24.8	(<.001)	(0.8)
Breast milk at discharge^f^	149	62.7	33.7	30.6	28.4	(<.001)	(<.001)	122	40.0	30.9	30.9	19.7	(0.02)	(0.04)

a) p value for bivariable analysis; b) adjusted for maternal age for pregnancy complications and caesarean section; p value adjusted for gestational age, small for gestational age and sex for infant outcomes. Small number of infants with brain lesions did not allowed calculation of aOR; c) <10th percentile of birthweight standards from the MOSAIC cohort; d) Hypertension during pregnancy: pregnancy-induced hypertension, pre-eclampsia, eclampsia and HELLP syndrome; e) In-hospital mortality includes death during labour, delivery or hospitalization after birth; f) Includes only infants discharge alive; g) Oxygen dependence or ventilation, including nasal continuous positive airway pressure, at 36 weeks of gestational age; h) Brain lesions includes intraventricular haemorrhage and periventricular leukomalacia; i) Long hospital stay: longer hospital stay than 75^th ^percentile for gestational age.

After adjustment for maternal age, gestational age, small for gestational age and sex, breastfeeding at discharge was lower in neighbourhoods with the highest unemployment rates (Trent: OR=0.2 (95% CI: 0.1-0.4); Ile-de-France: OR=0.4 (95% CI: 0.2-0.8)). The interaction between hypertension during pregnancy and region was not significant after adjustment. (interaction test p<0.07) (Additional file 1).

Neighbourhood socio-economic factors had a similar impact on the risk of very preterm singleton birth in two European regions with different health care systems and rates of very preterm birth. The risk of very preterm birth was doubled in the most deprived neighbourhoods compared to the least deprived. Consistent neighbourhood socio-economic gradients were not observed for any of the specific pregnancy or delivery characteristics for singleton very preterm births in the two regions and infant outcomes did not differ, with the exception of breastfeeding at discharge which was lower for infants living in more deprived neighbourhoods. These results support the hypothesis that the association between very preterm birth and socio-economic factors does not result from one single mechanism, but represents multiple disadvantages; these could include poor antenatal care, maternal stress, racism, poor health behaviours and biological factors, such as genetic predisposition, immune system changes or endocrine changes [11,30].

The strengths of our study are its use of a common protocol to abstract data from medical records and to ensure completeness of inclusions in a geographically-based cohort of very preterm infants. To our knowledge this study is the first to look at direct socio-economic comparisons of very preterm births between countries using standardised data collection. Nonetheless, the definitions of some data items were difficult to standardise in terms of their severity or thresholds for inclusion, in particular some pregnancy complications such as maternal haemorrhage, and this may explain some of the variability in rates between the regions. Regional level differences in definitions should not, however, affect the within region association with neighbourhood socio-economic factors.

Comparable data across countries on maternal socio-economic characteristics at the individual level were not routinely available from medical records in either region as is often the case in studies based on medical records or registers. Therefore, we used census data to assigned socioeconomic position to very preterm infants according to the place of residence of the mother. Census data also allowed us to obtain a comparison group of infants which was not available in our cohort of very preterm births and to study the distribution of births by neighbourhood socio-economic characteristics. Our study shows that this methodology can be used to assess socioeconomic inequalities in a cross-national context. This methodology could be used to study other health issues at an international level for which a comparison population, i.e. in terms of age or sex, can be obtained from the census. Census data have some limitations, however. Disadvantaged families may be undercounted in the census, which would lead to an overestimate of the impact of deprivation on very preterm birth. We verified that this was not a problem in our analysis by validating the deprivation quartiles from our census population with other data sources on births in both regions. Other limits of our comparison group (census infants) are that we were unable to exclude very preterm infants and multiples from the census population, as gestational age and multiplicity are not available in the census data; but these represent a small proportion of all live births and would thus have a very minor impact on the birth population deprivation quartiles. Infants who died in the first days of life will most likely not be included in the census, although these infants are a very small proportion of total births.

Our results were robust despite differences in the definitions used to report census data on socio-economic characteristics in England and France. The magnitudes of the risk of very preterm births for singletons in most deprived versus least deprived neighbourhoods were quite similar for Trent and Ile-de-France, and slightly higher than those reported previously in Europe [7,9] using deprivation scores. The measures chosen include components of validated area-level composite deprivation scores in the UK (the Carstairs score and the Townsend Index) and in France [31], although the scores do not include the same social characteristics (education is not included in the UK scores and home ownership is not included in the French score). Our study computed quartiles within each region, because of the differences in census definitions and since baseline values of our neighbourhood variables did not allow us to define common thresholds. The literature on neighbourhood socio-economic deprivation relies primarily on relative rather than absolute levels of deprivation. Use of relative deprivation measures may be less valid when inequality between regions is very different; however in the Trent and Ile-de-France, there were similar relative gaps between the lowest and the highest quartiles for the four socio-economic characteristics used. These various methodological challenges lead us to analyze the four variables separately and not attempt to develop a common deprivation score. However, given the consistency of our results across the indicators, a composite indicator looking at multi-dimensional deprivation would very likely give a similar or stronger effect. Further work would be needed, however, to validate this approach.

We found no systematic differences between pregnancy and delivery characteristics of very preterm births and neighbourhood of residence in the two regions. This finding corroborates previous studies which found no socio-demographic gradient for spontaneous delivery or PPROM for very preterm births in Australia [12] and no association of preterm birth with maternal complications (i.e. hypertension, preeclampsia and vaginal bleeding) during pregnancy in England [32]. In contrast, spontaneous very preterm deliveries in deprived neighbourhoods, particularly those before 29 weeks, have been associated with more fetal and maternal infections in the UK [15]. We found opposite associations between socio-economic characteristics and hypertension during pregnancy: lower rates among women living in deprived neighbourhoods in Trent and higher rates in Ile-de-France. Hypertensive complications have been reported to be more frequent among socially disadvantaged women [13,14]. In Ile-de-France, higher rates may be partly explained by higher proportions of women from sub-Saharan Africa who have been found to be at risk of hypertensive diseases during pregnancy and who live in poorer neighbourhoods [33]. It is not clear why rates were lower in Trent, but the distribution of maternal age might be an explanation if older women who are at higher risk of hypertension morbidity during pregnancy live in more affluent neighbourhoods. Another hypothesis could be under diagnosis of hypertensive diseases in women with inadequate antenatal care who live in less affluent neighbourhoods, such as young women.

Very preterm outcomes varied between the two regions, but they did not vary by neighbourhood socio-economic characteristics within each region, with the exception of breastfeeding. Similar results have been shown recently for survival, respiratory support, length of stay among very preterm infants [19] and the risk of cerebral palsy among very low birth-weight infants [34]. This suggests that once admitted to hospital equitable care is provided. Differences in breastfeeding at discharge may indicate, however, that socio-economic factors affect some aspects of care in the neonatal intensive care unit, in particular where parental involvement is required. Other studies have found that mothers of lower social class are less likely to breastfeed their high risk infants [35-37]. These mothers might experience greater difficulties establishing and maintaining lactation which requires acquiring knowledge about milk expression and storage procedures, having access to milk pumps and time for frequent expression and transport of milk to the neonatal unit. This difference also suggests that there may be differences in care after discharge and in longer term outcomes.

Despite the absence of an association between neighbourhood socio-economic status and infant mortality and morbidity, the total burden of mortality and respiratory and neurological morbidity will be greater in deprived areas because of their higher rates of preterm birth. In addition, the total burden of these socio-economic differences may be greater in England, where rates of very preterm births are higher, than in France [38]. The neonatal care load may also be greater in hospitals in deprived catchment areas.

## Discussion and conclusion

In conclusion, trends in the risk of very preterm birth according to area-level socio-economic characteristics were robust in two European regions with different health care systems and rates of very preterm birth leading to a greater health burden in deprived areas despite the absence of socio-economic disparities in mortality and short-term morbidity. Planning for neonatal care provision, social support services for families and follow-up programmes needs to consider the socio-spatial distribution of very preterm children. Extending the methodology used in this paper to other countries with access to area-based census data would make it possible to test whether these trends are similar in other settings.

### "What is already known on this topic"

1. Socio-economic disparities are associated with adverse obstetric and birth outcomes and an increased burden of neonatal care

2. Neonatal mortality and neonatal care for very preterm infants do not vary across area deprivation levels in the UK

3. Very preterm birth rates vary widely between European countries.

### "What this study adds"

1. Area deprivation more than doubled the risk of singleton very preterm birth, in two European regions with different health systems and differing rates of very preterm birth.

2. There were not consistent associations across the two regions in pregnancy complications and delivery characteristics by neighbourhood deprivation.

3. Area deprivation did not affect the outcomes to discharge from hospital for very preterm singletons with the exception of breastfeeding.

## Competing interests

The authors declare that they have no competing interests.

## Authors’ contribution

Analysis and interpretation of the data were carried out by MB, LS and JZ. MB wrote the first draft of the paper and all authors participated in revisions and approved the final manuscript. All authors read and approved the final manuscript.

## Pre-publication history

The pre-publication history for this paper can be accessed here:

http://www.biomedcentral.com/1471-2393/13/97/prepub

## Supplementary Material

Additional file 1: Adjusted odds ratio for maternal and infant's outcomes among very preterm singletons by neighbourhood unemployment rate quartiles. Click here for file
