# Supplementary material for: Neighbourhood deprivation and very preterm birth in an English and French cohort
Source: BMC Pregnancy Childbirth. 2013 Apr 25;13:97. doi: 10.1186/1471-2393-13-97 (PMC3640897; doi:10.1186/1471-2393-13-97)
Supplement: Additional file 1: — Adjusted odds ratio for maternal and infant's outcomes among very preterm singletons by neighbourhood unemployment rate quartiles. [file 1471-2393-13-97-S1.doc]

Appendix 1. Adjusted odds ratio for maternal and infant's outcomes among very preterm singletons by neighbourhood unemployment rate quartiles

|  | | | | | | | | | |
| --- | --- | --- | --- | --- | --- | --- | --- | --- | --- |
|  | **Unemployment rate quartiles** | | | | | | | | |
|  | **Trent** | | | |  | **Ile-de-France** | | | |
|  | **1st (least deprived)** | **2nd** | **3rd** | **4th (most deprived)** |  | **1st (least deprived)** | **2nd** | **3rd** | **4th (most deprived)** |
| **Characteristics of very preterm pregnancy and delivery** |  |  |  |  |  |  |  |  |  |
| Hypertension during pregnancy^a^ | 1 | 0.6 | 0.5 | 0.5 |  | 1 | 1 | 1.0 | 1.6 |
|  |  | (0.3-1.3) | (0.2-1.0) | (0.3-0.9) |  |  | (0.5-2.1) | (0.5-2.0) | (0.9-3.0) |
|  |  |  |  |  |  |  |  |  |  |
| Antepartum hemorrhage without hypertension | 1 | 1.2 | 1 | 1.2 |  | 1 | 0.8 | 0.7 | 0.8 |
|  |  | (0.7-2.2) | (0.6-1.7) | (0.7-2.1) |  |  | (0.4-1.7) | (0.4-1.4) | (0.4-1.6) |
|  |  |  |  |  |  |  |  |  |  |
| Premature rupture of membranes without hypertension or antepartum hemorrhage | 1 | 0.6 | 1 | 0.4 |  | 1 | 0.6 | 1.0 | 0.5 |
|  |  | (0.3-1.5) | (0.5-2.2) | (0.2-0.9) |  |  | (0.3-1.2) | (0.6-1.9) | (0.3-1.0) |
|  |  |  |  |  |  |  |  |  |  |
| Preterm labour without hypertension. hemorrhage or PPROM | 1 | 1.9 | 2 | 3.0 |  | 1 | 1 | 0.8 | 1.2 |
|  |  | (0.6-6.4) | (0.6-6.4) | (1.0-9.0) |  |  | (0.5-2.2) | (0.4-1.6) | (0.6-2.4) |
|  |  |  |  |  |  |  |  |  |  |
| Caesarean section before labour | 1 | 1.0 | 1.0 | 1.1 |  | 1 | 1.4 | 1.2 | 1.1 |
|  |  | (0.5-1.8) | (0.6-1.8) | (0.6-1.9) |  |  | (0.8-2.5) | (0.7-2.0) | (0.6-1.8) |
|  |  |  |  |  |  |  |  |  |  |
| **Very preterm infant's outcomes** |  |  |  |  |  |  |  |  |  |
| In-hospital mortality^b^ | 1 | 1.4 | 2.0 | 1.5 |  | 1 | 0.8 | 0.4 | 0.5 |
|  |  | (0.6-3.7) | (0.9-4.8) | (0.7-3.6) |  |  | (0.4-1.6) | (0.2-0.9) | (0.3-1.1) |
|  |  |  |  |  |  |  |  |  |  |
| Bronchopulmonary dysplasia^c,d^ | 1 | 1.4 | 0.8 | 1.1 |  | 1 | 1.6 | 1.7 | 1 |
|  |  | (0.6-3.5) | (0.3-1.9) | (0.5-2.4) |  |  | (0.5-4.7) | (0.6-4.7) | (0.3-2.9) |
|  |  |  |  |  |  |  |  |  |  |
| At least one day on ventilator^c^ | 1 | 1.3 | 1 | 0.8 |  | 1 | 0.9 | 1.0 | 0.7 |
|  |  | (0.6-2.7) | (0.5-1.9) | (0.4-1.4) |  |  | (0.4-2.0) | (0.5-2.1) | (0.3-1.5) |
|  |  |  |  |  |  |  |  |  |  |
| Long hospital stay^c,e^ | 1 | 1 | 0.6 | 1 |  | 1 | 0.8 | 0.8 | 0.7 |
|  |  | (0.5-2.0) | (0.3-1.2) | (0.5-1.8) |  |  | (0.4-1.7) | (0.4-1.6) | (0.4-1.4) |
|  |  |  |  |  |  |  |  |  |  |
| Breast milk at discharge^c^ | 1 | 0.3 | 0.2 | 0.2 |  | 1 | 0.6 | 0.7 | 0.4 |
|  |  | (0.1-0.5) | (0.1-0.5) | (0.1-0.4) |  |  | (0.3-1.3) | (0.4-1.4) | (0.2-0.8) |

aOR: adjusted for maternal age for characteristics of very preterm delivery ; p value adjusted for gestational age, small for gestational age and sex for infant outcomes. Small number of infants with brain lesions did not allowed calculation of aOR; a) Hypertension during pregnancy: pregnancy-induced hypertension, pre-eclampsia, eclampsia, and HELLP syndrome; b) In-hospital mortality includes death during labour, delivery or hospitalization after birth; c) Includes only infants discharge alive; d) Oxygen dependence or ventilation, including nasal continuous positive airway pressure, at 36 weeks of gestational age; e) Long hospital stay: longer hospital stay than 75^th^ percentile for gestational age.
